# The Cell Biology of Metastatic Invasion in Pancreatic Cancer: Updates and Mechanistic Insights

**DOI:** 10.3390/cancers15072169

**Published:** 2023-04-06

**Authors:** Vidhu B. Joshi, Omar L. Gutierrez Ruiz, Gina L. Razidlo

**Affiliations:** 1 Department of Biochemistry & Molecular Biology, Mayo Clinic, Rochester, MN 55905, USA; 2 Division of Gastroenterology & Hepatology, Mayo Clinic, Rochester, MN 55905, USA

**Keywords:** pancreatic cancer, metastasis, epithelial-to-mesenchymal transition, invadopodia, invasion, focal adhesions

## Abstract

**Simple Summary:**

Pancreatic cancer remains one of the most lethal forms of cancer, with high rates of metastasis. Metastasis is a complex, multi-step process involving many dynamic changes in cellular shape, motility, and adhesion, which cannot be captured by most analyses of bulk tumors. An improved understanding of the cell biological processes that occur during tumor cell invasion and dissemination in pancreatic cancer is needed in order to work towards inhibiting metastasis as a component of cancer therapy.

**Abstract:**

Pancreatic ductal adenocarcinoma (PDAC) is one of the leading causes of cancer-related mortality worldwide. This is largely due to the lack of routine screening protocols, an absence of symptoms in early-stage disease leading to late detection, and a paucity of effective treatment options. Critically, the majority of patients either present with metastatic disease or rapidly develop metastatic disease. Thus, there is an urgent need to deepen our understanding of metastasis in PDAC. During metastasis, tumor cells escape from the primary tumor, enter the circulation, and travel to a distant site to form a secondary tumor. In order to accomplish this relatively rare event, tumor cells develop an enhanced ability to detach from the primary tumor, migrate into the surrounding matrix, and invade across the basement membrane. In addition, cancer cells interact with the various cell types and matrix proteins that comprise the tumor microenvironment, with some of these factors working to promote metastasis and others working to suppress it. In PDAC, many of these processes are not well understood. The purpose of this review is to highlight recent advances in the cell biology of the early steps of the metastatic cascade in pancreatic cancer. Specifically, we will examine the regulation of epithelial-to-mesenchymal transition (EMT) in PDAC and its requirement for metastasis, summarize our understanding of how PDAC cells invade and degrade the surrounding matrix, and discuss how migration and adhesion dynamics are regulated in PDAC to optimize cancer cell motility. In addition, the role of the tumor microenvironment in PDAC will also be discussed for each of these invasive processes.

## 1. Introduction

Pancreatic cancer is the seventh leading cause of cancer-related deaths worldwide and is predicted to become the third leading cause of cancer-related deaths by 2025 [1], with a 5-year overall survival rate of approximately 12% [2]. Histologically, malignancy of the exocrine pancreas—pancreatic ductal adenocarcinoma (PDAC)—constitutes over 90% of pancreatic cancer cases [3]. Tumorigenesis in PDAC is largely driven by distinct genetic mutations that collectively drive oncogenesis. These insults lead to the development of pancreatic intraepithelial neoplasias (PanIN), which can progress to PDAC [4,5,6]. The progression of PanIN lesions to PDAC is also associated with desmoplastic changes in the tumor stroma that can promote tumor growth, enhance disease progression, and confer resistance to therapy [7,8,9].

Clinically, over 80% of patients present with locally advanced unresectable disease or metastatic disease [10,11]. Critically, evidence suggests that PDAC cells can acquire pro-metastatic traits and disseminate very early in disease progression, even prior to the formation of the primary tumor [12]. This might help explain what is observed clinically, wherein up to 30% of patients who undergo surgical resection with negative microscopic margins demonstrate distant metastases within a year of surgery [13]. This suggests that metastasis in PDAC is an ongoing process that begins early, where a subset of tumor cells disseminates prior to detection. Taken together, the dismal outcomes and the absence of effective treatment options point to an urgent need to detect and treat oligometastatic disease. Achieving this goal requires improving our understanding of the biological processes driving metastasis in PDAC.

Even if PDAC patients have already developed metastatic disease, it is still essential and clinically relevant to understand the metastatic process with the goal of therapeutic targeting to block further dissemination. First, metastasis is an ongoing process, with both the primary and secondary tumors seeding new metastatic tumors, and even reseeding the primary tumor [14]. Thus, halting this ongoing metastatic spread would slow disease progression. Second, cells that are actively metastasizing may be less sensitive to cytotoxic therapies. Migration and proliferation are thought to be mutually exclusive, known casually as the “go or grow” effect, and quiescence is hallmark of both chemoresistant and disseminating metastatic cells. Thus, actively invading cells may be resistant to chemotherapeutic agents that typically target proliferating cells, and inhibiting metastatic invasion may re-sensitize tumor cells to cytotoxic therapies [15,16]. Finally, some anti-cancer therapies, including surgery, radiation, and some chemotherapies, can actually upregulate the invasive machinery in residual tumor cells [17,18]. While quite understudied, potential mechanisms include direct activation of invasive signaling, activation of macrophages that promote tumor cell invasion, amplified inflammatory signaling, hypoxia, and mechanical disruption. Thus, targeting metastatic invasion in the context of conventional therapies could minimize metastatic spread and improve outcomes. The challenge is understanding the mechanics of metastasis in this disease and identifying druggable targets.

However, at the present time, a comprehensive model of the cell biology of metastasis in PDAC does not exist. While genetic studies have been instrumental in identifying genetic aberrations in metastatic versus primary tumors [19], they do not enable us to build a functional model of how primary PDAC cells accomplish metastatic invasion. The highly dynamic processes of invasion and migration are composed of transient cellular changes in protein activation, localization, and post-translational modifications. A model of PDAC metastasis must also define the role of the tumor microenvironment (TME) in this process. In PDAC, the TME is heterogeneous and contains cell types that can either promote or suppress metastatic invasion such as myeloid-derived suppressor cells, tumor-associated macrophages, regulatory T cells, B lymphocytes, pancreatic stellate cells (PSCs), and cancer-associated fibroblasts (CAFs) [20,21]. By improving our understanding of the TME’s role in PDAC metastasis, we can enable the development of therapeutic strategies that target components of the TME to prevent or halt metastasis.

**Figure 1:**
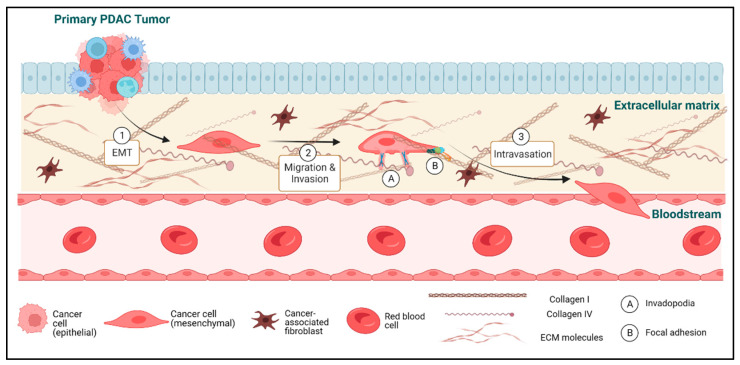
Early metastatic cascade in PDAC. (1) Epithelial-to-mesenchymal transition (EMT) is one of the hallmarks of metastasizing tumor cells. EMT is associated with several molecular and morphological changes, including loss of cell–cell adhesions and apico-basal polarity and reduced proliferation, as well as the upregulation and downregulation of several mesenchymal (e.g., N-cadherin) and epithelial (e.g., E-cadherin) markers, respectively. (2) Metastasizing tumor cells migrate and invade the surrounding extracellular matrix (ECM) either alone or in cell clusters. PDAC is associated with a strong desmoplastic reaction that can both promote and inhibit cancer cell migration and invasion. PDAC cells rely on structures such as invadopodia to invade and degrade the surrounding ECM, while using structures such as focal adhesions to adhere and move through the ECM. Focal adhesions rely on integrin-mediated interactions with the proteins that comprise the ECM, including collagens and fibronectins. (3) The final step in the early metastatic cascade involves the extravasation of the tumor cells into the bloodstream or lymphatics, through which they can travel in circulation and potentially form distant metastases elsewhere in the body. Figure created on BioRender.com.

Metastasis is a complex, multi-step process that includes detachment of tumor cells from the primary tumor, acquisition of a migratory phenotype, invasive degradation of the extracellular matrix (ECM) and basement membrane to support dissemination, intravasation and survival in the circulation, extravasation, and, finally, survival and proliferation in a secondary site with a distinct microenvironment. Failure at any of these steps would prevent the outgrowth of metastatic tumors. The purpose of this review is to summarize recent advances in our mechanistic knowledge of the earliest steps of metastatic dissemination in PDAC, including cancer cell migration and invasion. These processes involve epithelial-to-mesenchymal transition (EMT), the invasive degradation of the ECM through formation of structures called invadopodia, and the adhesive movement of cancer cells within the extracellular matrix via the activity of lamellipodia and focal adhesions (Figure 1). This review will also discuss the role of the TME in each of these processes, key questions raised, and future directions in the investigation of metastasis in PDAC.

## 2. Epithelial-to-Mesenchymal Transition in PDAC

The epithelial-to-mesenchymal transition (EMT) is a developmental process that is widely viewed as an early step in metastasis. During EMT, epithelial cells transition to a more mesenchymal-like state that is broadly characterized by loss of apico-basal polarity, loss of epithelial cell-to-cell adhesions, increased migratory capacity, increased resistance to cell death, morphological changes, and a less proliferative state [22]. These changes are advantageous to cancer cells in the early steps of metastatic dissemination, as the cells become more capable of migrating and invading the local microenvironment [23]. EMT in cancer is generally marked by transcriptional downregulation of epithelial markers, including E-cadherin, and upregulation of mesenchymal markers, including N-cadherin and vimentin, via activity of the transcription factors, including Twist-1, Snail, and ZEB1 [22]. EMT has widely been acknowledged as a critical step in metastatic dissemination. However, recent evidence has challenged the importance of EMT in the metastasis of certain cancer types [24,25,26]. Furthermore, new findings indicate that cells undergo EMT on a spectrum of transient, intermediate states which form through complex intracellular processes and interactions with the local microenvironment, with the specific molecular changes and accompanying intermediate states being tumor specific [27]. These findings highlight the importance of uniquely defining the EMT spectrum, characterizing its key regulators, and examining whether EMT is a critical step early in the metastatic cascade for each cancer type. Here, we will examine the importance of EMT in metastasis, discuss key mechanistic insights into the action of EMT in PDAC through downregulation of E-cadherin, and explore the role of the PDAC tumor microenvironment in EMT.

### 2.1. The Complex Role of EMT in Metastasis

EMT is widely associated with metastasis, and transcriptional changes that are hallmarks of classical EMT are often even used as a surrogate readout for metastatic capacity. In PDAC, mouse models revealed that EMT occurs extremely early in disease progression in disseminating tumor cells, even before the development of a primary tumor [12]. In contrast, intriguing recent studies have suggested that metastasis can occur in the absence of EMT [24,25,28], and that canonical EMT factors may promote tumor progression by EMT-independent pathways [29]. For example, Zheng reported that EMT is not required for invasion or metastasis in PDAC [30]. Genetic loss of EMT transcription factors Snail or Twist did not inhibit tumor cell invasion and dissemination in the circulation, and had no effect on the number of distant metastases in a genetic mouse model of PDAC [30]. This is similar to a study that showed that inhibiting EMT did not prevent the formation of metastases in a breast cancer model, and lung metastases derived from primary breast tumors maintained their epithelial phenotype [26]. Given the long-standing belief in the critical role of EMT in cancer metastasis, these studies generated controversy [31,32]. Debate has focused on whether these studies used adequate definitions and markers of EMT, and whether EMT was suppressed in its entirety.

To address this debate, recent evidence suggests that EMT is likely a dynamic spectrum of partial states between the complete epithelial and mesenchymal phenotypes—rather than two static, binary fates of a cancer cell [33,34]. These partial EMT (pEMT) states—wherein cancer cells acquire some mesenchymal phenotypic traits while maintaining other epithelial traits—have been captured in multiple malignancies, including lung adenocarcinoma [35], breast cancer [36], clear cell renal cell carcinoma (ccRCC) [37], ovarian cancer [38], and skin squamous cell carcinoma [39]. In PDAC, Carstens et al. reported distinct phenotypic clusters across the EMT spectrum in human and mouse PDAC samples using single-cell RNA sequencing, including clusters that were distinctly epithelial- or mesenchymal-like, as well as predominant pEMT [40]. In addition to transcriptional pEMT, canonical EMT factors can also be regulated post-transcriptionally to affect their protein levels or function, as discussed below. In addition to highlighting heterogeneity among PDAC cells, these findings also illustrate the lack of a singular definition of an EMT phenotype.

It is unknown whether a partial EMT phenotype confers greater metastatic potential compared to “complete” transcriptional EMT. One way that a partial or transient EMT phenotype may confer metastatic benefits is by altering the cellular mode of migration. While EMT-enabled metastasis often indicates single disseminating cancer cells that maintain a mesenchymal-like phenotype, which has been observed using intravital imaging in a mouse model of PDAC [41], cancer cells are also capable of metastatic dissemination in cell clusters wherein they maintain cell-to-cell adhesions, referred to as collective migration [36,42,43,44,45,46,47]. In PDAC, a 3D histological analysis of the tumor-host interface suggested that single cell invasion was exceedingly rare (<1%) compared to collective migration [48]. Collective migration has been observed in PDAC cells lacking Snail and Twist, which have a stable epithelial phenotype [40], and in cells with only a partial downregulation of E-cadherin (discussed further below). In addition to collective migration, invasion in the absence of EMT may also occur by distinct modes of cell migration, including amoeboid-style blebbing, which does not require a mesenchymal phenotype or proteolytic degradation of the ECM [49,50]. Instead, amoeboid-style migration utilizes acto-myosin contractility, and can result in very fast migration in vivo [51,52]. Critically, cancer cell migration is highly plastic and heterogenous, and tumor cells can rapidly switch among modes of migration depending on their environment [53,54]. Thus, maintaining a partial EMT phenotype may enable tumor cells to rapidly adapt to changes in the microenvironment in order to facilitate migration and survival.

Together, these findings demonstrate the heterogeneity found within metastasizing cancer cells with respect to the expression of epithelial and mesenchymal markers, and modes of cell migration, thus underscoring the need for high-resolution, single-cell protein-level analyses when studying EMT and metastatic dissemination. Future in vivo studies may identify phenotypes on the epithelial-mesenchymal spectrum that are associated with greater migratory, invasive, and metastasis-forming capabilities. Furthermore, characterizing these phenotypes in patient-derived samples is essential to determining whether they are recapitulated PDAC patients, with the limitation that the transient nature of such changes in rare migrating cells is difficult to capture in fixed samples. Crucially, the complexity of EMT indicates that simply using EMT transcript or protein expression is not a sufficient indicator of metastatic capacity.

### 2.2. Novel Mechanistic Insights on EMT in PDAC: E-Cadherin

EMT in PDAC is regulated by a host of transcription factors, inflammatory cytokines, non-coding genetic elements, epigenetic changes, and extracellular signals from other cell types in the TME, which has been extensively reviewed elsewhere [28] A limitation within the PDAC EMT literature is the paucity of specific mechanisms of EMT, rather than simply using EMT hallmark gene/protein expression as a descriptor for metastatic capacity. This section focuses on novel mechanistic insights of the functional consequences of EMT in PDAC by focusing on a canonical EMT protein, E-cadherin.

One of the principal hallmarks of EMT in cancer is the downregulation of the epithelial-cadherin (E-cadherin) gene (CDH1) [55]. E-cadherin plays a critical role in regulating cellular adhesion between epithelial cells through the formation of multi-protein complexes at adherens junctions at the cell surface [56]. Downregulation of E-cadherin leads to a loss of cell-to-cell adhesions, enabling cancer cells to detach from the primary tumor in order to permit invasion and metastatic dissemination [22].

The classical definition of EMT includes transcriptional downregulation of E-cadherin. In PDAC, E-cadherin expression can be regulated both transcriptionally and epigenetically to enhance metastasis in vivo [57]. In PDAC, the transcriptional repressor Snail (SNAI1) co-localizes with histone deacetylases 1 and 2 (HDAC1 and HDAC2, respectively) at the CDH1 promoter in PDAC cells, resulting in markedly reduced E-cadherin expression and high in vivo metastatic potential [57]. Similarly, binding of the transcription factor ZEB1 to the promoter of CDH1 increased the binding of HDAC1 and HDAC2 to CDH1, and inhibition of HDAC 1 and HDAC 2 or siRNA-mediated knockdown of ZEB1 significantly reduced the migratory capacity of PDAC cells in vitro [58]. These studies highlighted how transcriptional repression and deacetylation of the CDH1 gene downregulate E-cadherin, driving PDAC cells to a more migratory state with enhanced metastatic potential.

Importantly, transcriptional repression and epigenetic silencing are not the only mechanisms by which PDAC cells downregulate E-cadherin activity. E-cadherin internalization, trafficking, and lysosomal degradation are mechanisms by which epithelial cell adhesion can be modulated even without transcriptional downregulation of E-cadherin [59]. While the internalization of E-cadherin and its subsequent vesicular trafficking have long been known to regulate its function, a key study by Aiello et al. indicated a post-transcriptional mechanism used by PDAC cells to downregulate E-cadherin activity and promote pEMT [47]. E-cadherin can be internalized and sequestered in endocytic recycling vesicles via the Rab11 GTPase, and this internalization also supports morphological changes to a more mesenchymal-like state, independent of transcriptional downregulation [47]. As suggested above, these cells disseminate from the primary tumor in cell clusters, where, notably, the E-cadherin protein was found at cell–cell junctions and not on the cluster surface [47]. This junctional expression supports cell–cell adhesion and survival in the circulation. This partial retention of epithelial characteristics also promotes metastatic colonization at secondary sites by allowing rapid trafficking of E-cadherin back to the cell surface and adaptation of a more epithelial morphology. This was also shown by Carstens and colleagues, who found that an epithelial phenotype in PDAC cells supported collective migration and metastatic outgrowth in the liver, and that the uncoupling of the gene expression and protein levels of E-cadherin indicates post-transcriptional regulation may be key in the dynamic regulation of E-cadherin function [40]. This is impactful because it would allow for a rapid and transient sequestration and reversible membrane localization of E-cadherin. Similarly, in other cancer types, retention of E-cadherin expression allows for the clustering or collective migration of groups of adherent cells, or for the rapid conversion back to an epithelial morphology and formation of cell–cell attachments to establish secondary tumors [60,61]. Thus, while transcriptional downregulation of E-cadherin may not be required for metastasis via canonical EMT, the intracellular distribution and trafficking of E-cadherin may functionally accomplish the same goal.

In addition to its internalization, the extracellular region of E-cadherin can be cleaved by extracellular proteases, resulting in a soluble E-cadherin fragment [62]. While the presence of soluble E-cadherin has been considered as a biomarker, shedding of the extracellular domain of E-cadherin also has functional consequences, including disrupting cell adhesions, activating survival signaling, and promoting migration [63]. In pancreatic cancer cells, the elevated expression of the protease kallikrein 7 can cleave E-cadherin, resulting in decreased cell association and increased invasion [64]. Together, these findings indicate the complex regulation of E-cadherin in pancreatic cancers and its critical role in promoting tumor cell invasion and survival.

### 2.3. Role of the TME in EMT

The PDAC TME is composed of multiple cell types that modulate the anti-tumor immune response, disease progression, and response to therapy through a complex web of interactions with the primary tumor [20,21,65]. Multiple cell types in the PDAC TME promote classical (transcriptional) tumor cell EMT, including tumor-associated macrophages, T cells, pancreatic stellate cells, and stromal myofibroblasts [66,67,68,69,70]. For example, the CD4+ T-helper cell-derived cytokine interleukin 22 (IL22) promotes PDAC formation, EMT, and metastasis [67,71]. Furthermore, other pleiotropic inflammatory cytokines in the TME have been implicated in promoting EMT, such as transforming growth factor-β (TGF-β), tumor necrosis factor-α (TNF-α), IL1, and IL6 [28]. Collectively, these findings suggest a major role for inflammatory signaling in the TME as a promoter of EMT in PDAC.

Interestingly, recent evidence also suggested that components of the TME downregulate EMT and disease progression. Specifically, Özdemir et al. studied the role of stromal myofibroblasts in disease progression and metastasis. Depletion of αSMA+ myofibroblasts in the KTC (Ptf1a-Cre/+;LSL-KrasG12D/+;Tgfbr2flox/flox) mouse model of PDAC [70,72] actually promotes EMT and tumorigenesis, and leads to poorer survival [70].This study challenged prior evidence suggesting that the αSMA+ myofibroblasts in the PDAC tumor stroma are pro-tumorigenic and are responsible for disease progression [73]. In this regard, collective evidence regarding stromal fibroblasts in PDAC now suggests the presence of different subtypes of stromal fibroblasts that are heterogeneous with respect to their influence on the tumor (i.e., tumor-promoting versus tumor-restraining) [74].

These studies suggest that the TME can both promote and suppress EMT in PDAC. While current studies on individual components of the TME further our understanding of how each cell type regulates EMT, future studies should focus on elucidating how these TME components interact with each other, and how these interactions influence the EMT spectrum within the primary tumor. One of the major challenges associated with studying the TME in PDAC is recapitulating the TME, as well as capturing the dynamic, multi-directional interactions occurring between the tumor and TME in an experimental setting. In this regard, in vivo studies wherein the TME can be best recapitulated offer the preferred experimental platform for uncovering these interactions within the TME.

## 3. Invadopodia and Protease-Mediated Degradation of the ECM

Following EMT and detachment from the primary tumor through loss of cell–cell adhesions, tumor cells navigate across the basement membrane and through the dense tumor stroma. In order to accomplish this, tumor cells can form structures called invadopodia, which are actin-based protrusions that promote cancer cell invasion through proteolytic degradation of the ECM [75]. The assembly, maturation, and function of invadopodia are a cascade of events that tightly regulate the signaling, localization, and function of multi-protein complexes, both intracellularly and at the site of invadopodia formation [76]. These dynamic protrusions harbor complexes composed of F-actin, actin regulatory proteins, kinases, adaptors, Rho GTPases, proteases, integrins, and other molecules that regulate invadopodia formation and activity [77,78].

Invadopodia have been observed in cell and 3D spheroid models, as well as in human PDAC tumors [79,80,81,82]. In vitro studies have demonstrated that invadopodia enable cancer cell invasion, and thus play a major role in the early metastatic cascade [83]. However, in vivo evidence of invadopodia is limited, due in part to technical limitations that prevent high resolution and real-time visualization of these dynamic structures in animal models [77]. In an analysis of human PDAC tumor samples, Chen et al. identified invadopodia-like structures in 45% (5/11) of tumors, emphasizing the clinical relevance of invadopodia in this disease context [79].

There are still gaps in the understanding of processes, both intratumorally and in the TME, that stimulate and regulate invadopodia function in PDAC. In this section, we provide an overview of our current understanding of invadopodia-mediated ECM degradation in PDAC as well as mechanistic insights on invadopodia formation and their regulation by the TME.

### 3.1. Novel Mechanistic Insights on Invadopodia in PDAC

**Figure 2:**
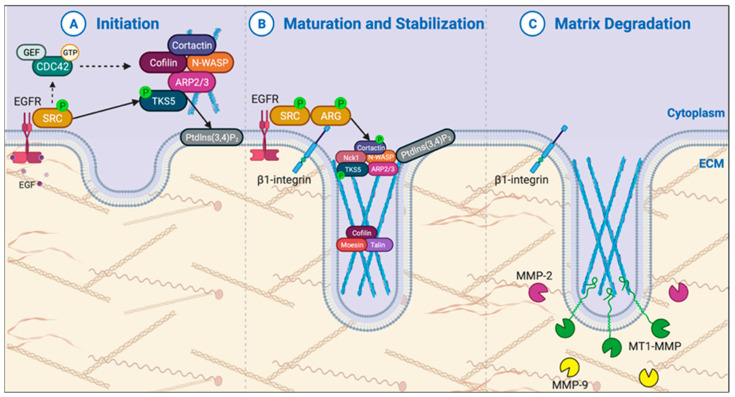
The initiation and assembly of functional invadopodia. (**A**) Invadopodia can be initiated by several intra- or extracellular cues (e.g., growth factor stimulation). Following this, Src kinase is autophosphorylated, leading to downstream activation of Cdc42 via a GEF (such as Vav1), phosphorylation of the scaffold protein TKS5, and formation of the invadopodia precursor core complex. The precursor core complex adheres to the membrane by binding to membrane-bound phosphatidylinositol-3,4-bisphosphate (PtdIns(3,4)P_2_). (**B**) The maturation and stabilization of invadopodia involves multiple steps, including: (i) activation of β1-integrin, the tyrosine kinase Arg, and cofilin; (ii) recruitment of proteins such as Nck1, moesin, and talin; and (iii) actin polymerization (blue filaments in figure). (**C**) Mature invadopodia are identified by their ability to degrade the surrounding matrix via matrix metalloproteinases (MMPs), such as MT1-MMP, MMP-2, and MMP-9. The mechanisms by which each of these MMPs is regulated and released into the ECM are unique and complex; they include exocytosis, endocytosis, and other intracellular transport mechanisms. (EGFR—epidermal growth factor receptor; GEF—guanine nucleotide exchange factor; GTP—guanosine triphosphate). Figure created on BioRender.com.

Formation of functional invadopodia can be divided into three parts: (1) invadopodia initiation and formation, (2) invadopodia maturation and stabilization, and (3) invadopodial degradation of the ECM (Figure 2). In this section, we will review insights into the regulation of each of these steps in PDAC.

#### 3.1.1. Invadopodia Initiation & Formation

The general process of invadopodia initiation has been described in other tumor cell models and includes a biochemical stimulus that activates the potent oncogenic kinase Src, which then phosphorylates multiple downstream substates resulting in recruitment and assembly of the actin nucleation machinery complex (N-WASP, Arp2/3, Cofilin) (Figure 2A) [84]. Generation of phosphatidylinositol-3,4-bisphosphate (PtdIns(3,4)P_2_) at the plasma membrane recruits key invadopodial components [85]. Activation of the small GTPase CDC42 then activates actin nucleation to initiate invadopodia formation [86]. CDC42 is activated by guanine nucleotide exchange factors (GEFs) and inhibited by guanine nucleotide-associated proteins (GAPs), with active, GTP-bound CDC42 binding effectors to regulate actin dynamics.

In pancreatic cancers, most tumors are driven by activating mutations in the oncogene KRAS. In addition to its well-known role in promoting tumor cell proliferation, KRAS signaling also drives invadopodia formation via multiple effector pathways, including downstream MEK/ERK2 signaling [81] and through the RalB GTPase and its effector RalBP1 via its ATPase function [87]. However, in both studies, inhibition of mutant KRAS signaling failed to completely abrogate invadopodia formation, suggesting there are multiple mechanisms that activate invadopodia formation in PDAC.

The potent kinase Src is a master regulator of invadopodia formation, as it phosphorylates multiple substrates to drive invadopodia assembly and function. In PDAC, Src activity is frequently amplified by multiple mechanisms, including dysregulated growth factor signaling. Once activated, essential invadopodial Src substrates in PDAC include the actin-binding protein cortactin, the scaffold TKS5, and the CDC42 GEF Vav1 [79,88,89]. Depletion of TKS5, which is recruited early in invadopodium assembly, significantly reduces invadopodia formation in PDAC cells [76,79]. In addition, Src mediates activation of the RET tyrosine kinase and leads to its co-localization and binding to TKS5 to promote invadopodia formation via the adaptor protein GRB2 [90]. Intriguingly, Src-mediated phosphorylation of the GEF and proto-oncogene Vav1, which is aberrantly expressed in a subset of pancreatic cancers and activates CDC42, appears to be dominant in invadopodial matrix degradation. A phospho-mimetic mutant of Vav1 aggressively promoted invadopodia formation, even in the presence of a Src inhibitor [88]. Together, these studies emphasize the critical role that Src plays in invadopodia formation and the recruitment of key proteins in PDAC.

Activation of CDC42 leads to the recruitment of N-WASP, which is required for invadopodia through recruiting the actin-nucleating Arp2/3 complex [91,92]. In PDAC, low levels of N-WASP correlate with improved survival, and genetic knockout of N-WASP inhibits tumor development in a mouse model of PDAC [93,94]. In addition to its invadopodial function, N-WASP also contributes to the cell biology of PDAC invasion by promoting migration in response to a chemotactic gradient [95]. Similarly, components of the Arp2/3 complex are also highly expressed in PDAC cells and promote migration, as well as invadopodial degradation [96].

#### 3.1.2. Invadopodia Maturation & Stabilization

Invadopodia stabilization involves anchoring the invadopodial actin filaments to the plasma membrane and ECM, as well as actin polymerization to protrude into the ECM. These processes involve the activity of integrins, tyrosine kinases, adaptor proteins, proteins that mediate actin polymerization, and proteins that bundle and cross-link actin [76]. However, there is minimal understanding of invadopodia stabilization in PDAC.

In PDAC, the actin-bundling protein fascin, which stabilizes invadopodial actin and promotes ECM degradation, is highly expressed and is associated with poor outcome [97,98]. Similarly, the actin-binding and cross-linking protein α-actinin 4 is elevated in PDAC and supports invadopodial degradation [99]. Invadopodia lifetime and stability is regulated by a direct interaction between α-actinin 4 and the large GTPase dynamin 2 (Dyn2), thereby promoting ECM degradation and invasion [100]. Dyn2 and α-actinin 4 are both overexpressed in PDAC, they directly interact and co-localize at invadopodia [100]. Dyn2 itself has also previously been implicated in invadopodia formation and invasive PDAC cell migration [101,102]. Finally, the formation of podosomes, which are highly related to invadopodia, is regulated in PDAC cells by an interaction between the actin-binding protein cortactin and the protein ezrin, which links the actin cytoskeleton and plasma membrane [103].

Some of the major challenges in studying invadopodia stabilization are technical limitations that make it difficult to capture this intermediate state between invadopodia formation and maturation. Thus, the lifespan of invadopodia can be used as an indirect experimental readout of invadopodia stability. However, multiple studies have demonstrated that invadopodia are highly dynamic, and that invadopodia turnover is critical in maintaining an effective invasive phenotype as the cell degrades and migrates through the ECM, and that increased stability can even reduce migratory capabilities [104,105,106,107]. Therefore, further studies on invadopodia stability and turnover should also examine the degradative ability of the invadopodia in tandem.

#### 3.1.3. Invadopodia Proteinase Activity

Functional invadopodia are characterized by their ability to degrade the ECM via proteinase activity [76]. Matrix metalloproteinases (MMPs) membrane-type I matrix metalloproteinase (MT1-MMP, also called MMP14), matrix metalloproteinase-2 (MMP-2), and matrix metalloproteinase-9 (MMP-9) are essential for invadopodia-mediated ECM degradation [108]. MT1-MMP is the only membrane-anchored MMP, and it is specifically recruited to and functions at invadopodia. Invadopodial MT1-MMP can degrade the ECM, as well as activate additional proteases, including MMP2, and signaling molecules [109]. While MMP expression is broadly studied in PDAC, here we will focus on the specific invadopodial recruitment and activity of MMPs.

*MT1-MMP.* MT1-MMP is regulated via multiple processes, including its transcription, endocytosis, exocytosis, and phosphorylation [108,109]. In PDAC, MT1-MMP expression is regulated in part by CD44 signaling, which upregulates the transcription factor Snail, leading to increased MT1-MMP expression and enhanced matrix degradation in vitro [110,111]. Newly synthesized MT1-MMP is targeted to invadopodia via vesicle trafficking pathways. Thereafter, it is internalized via clathrin-mediated endocytosis, where it can subsequently be recycled and trafficked back to the cell surface. In PDAC, oncogenic KRAS has been shown to interact with the scaffold IQGAP1 and the small G protein ARL4C to regulate the membrane recruitment of MT1-MMP in order to support ECM degradation and invasion [112]. Disruption of the endocytic internalization and trafficking of MT1-MMP impacts ECM degradation and invasion by modulating the amount of MT1-MMP on the cell surface [113]. Once trafficked to the plasma membrane, MT1-MMP is stabilized at invadopodia through a direct interaction with actin [114]. In PDAC, a potent regulator of the MT1-MMP/actin interaction is the membrane-type I matrix metalloproteinase C-terminus binding protein 1 (MTCBP-1). MTCBP-1 directly binds to the C-terminus of MT1-MMP and disrupts the interaction between MT1-MMP and F-actin, destabilizing MT1-MMP at invadopodia, and significantly reducing invadopodial lifetime, matrix degradation, and metastases [114,115,116]. MTCBP1 was highly expressed in primary pancreatic tumors, but had decreased expression in metastases, consistent with a role as a suppressor of invadopodia-mediated metastasis in PDAC cells.

*MMP-2.* MMP2 is a soluble protease released in an immature form containing a pro-domain, which is cleaved by MT1-MMP to yield an active, matrix-degrading protease. MMP-2 expression has been shown to be regulated in PDAC by glycogen synthase kinase 3β (GSK3β) by upregulating focal adhesion kinase (FAK) phosphorylation and active Rac1 (Rac1-GTP) [117]. Moreover, glutathione peroxidase 2 (GPX2) and tetraspanin 1 (TSPAN1) have both been shown to promote MMP-2 expression in PDAC cells, although the direct mechanistic links are unclear [118,119]. In hypoxic conditions, which are a common feature of PDAC [120,121], MMP-2 transcription is regulated by hypoxia-inducible factor 1-alpha (HIF-1α) and the actin-bundling protein Fascin via ERK2 phosphorylation [122].

*MMP-9.* In PDAC, there is limited evidence to help explain how MMP-9 secretion is regulated at sites of matrix degradation. MMP9 transcription is regulated in part through a miR-142-5p, which targets PIK3CA and suppresses the activation of the PI3K/AKT signaling pathway to reduce MMP9 [123]. However, previous studies have reported regulators of MMP expression that do not affect the invasive capability of cancer cells [111,124]. Thus, functional studies are critical to identify key regulators of MMPs and matrix degradation. In 2010, Miyazawa et al. observed that in PDAC, phosphorylated CUB domain-containing protein 1 (CDCP1) promotes secretion of MMP9 via a complex with protein kinase C delta (PKCδ), causing a significant increase in cancer cell invasion. Consistent with these findings, CDCP1 is correlated with poor prognosis in pancreatic cancer [125].

Overall, further studies are required to better understand how MMPs are recruited to invadopodia in PDAC, how MMP localization at sites of invadopodia is stabilized, and whether MMP signaling also plays a role in invadopodia stability and turnover.

### 3.2. Role of the TME in Invadopodial ECM Degradation

As described previously, PDAC cells and the associated TME are engaged in a complex network of bidirectional interactions that can influence the early steps of the metastatic cascade. For example, PDAC cells promote the activation of stromal fibroblasts, and activated fibroblasts signal to tumor cells both directly and through changes in the ECM [126]. There is emerging evidence that pancreatic stellate cells (PSCs), and their activated counterparts, cancer-associated fibroblasts (CAFs), promote invadopodial ECM degradation by tumor cells. Co-culture of PDAC cells with PSCs promotes PSC differentiation, leading to a marked increase in the number of PDAC cells forming invadopodia, and resulting in a significant increase in the number of PDAC cells invading the surrounding extracellular matrix in a 3D culture system [80].

Recent evidence suggests that invadopodia formation and ECM degradation by PDAC cells might be facilitated by MMPs released by stromal fibroblasts. Stromal fibroblasts release inactive MMP-2 (pro-MMP-2) that is activated by cancer-cell-derived MT1-MMP, resulting in significantly enhanced invadopodia formation and matrix degradation, even in tumor cell lines that, independently, are incapable of invadopodial matrix degradation [127]. These data provide further evidence that the crosstalk between PDAC cells and stromal fibroblasts promotes tumor cell invasion.

In addition, active fibroblasts can also directly participate in ECM degradation via invadopodia-dependent and invadopodia-independent mechanisms. Brentnall et al. demonstrated that palladin-induced activated human myofibroblasts formed invadopodia-like structures in vitro and degraded the matrix [126,128]. The palladin-induced activated fibroblasts also created tunnels in vitro that co-cultured PDAC cells used to migrate markedly further than PDAC cells cultured alone [126]. In contrast, Cao & Eppinga et al. demonstrated that CAFs can also accomplish invasive matrix degradation through unique, non-invadopodial patterns of matrix degradation in vitro. This stromal-based degradation did not require Src, Dyn2, or Cdc42 function, which are essential in invadopodia formation [129]. This may be due in part to altered expression or trafficking of MT1-MMP. Future studies should further elucidate the machinery involved in the formation, stabilization, and function of the structures that facilitate invadopodia-independent ECM degradation.

Along this line, differential expression or activation of MMPs between tumor cells and stromal fibroblasts contributes to the cooperation between these compartments. For example, fibroblast growth factor-10 (FGF10), released by stromal cells, upregulated MT1-MMP mRNA expression in PDAC cells [130]. In addition, given the dense desmoplasia associated with PDAC, studies have also examined the impact of matrix stiffness and cellular contractile forces on the expression of MT1-MMP by PDAC cells, leading to differences in spread and contractility [131]. While the intracellular signaling mechanisms that modulate MT1-MMP protein levels in this context are unclear, these findings suggest an important role for ECM-induced mechanical forces as a potential regulator of MMP expression and activity.

PSCs have also been identified as a source of MMP-2 and MMP-9 in vitro and in vivo [132]. Pro-MMP-2 released by stromal fibroblasts can be cleaved and activated by cancer-cell-derived MT1-MMP [127]. In this regard, Koikawa et al. showed that PSCs facilitate degradation of the basement membrane (BM) and cancer cell invasion in 3D in vivo co-culture via expression of pro-MMP-2 and its activator, MT1-MMP [133]. MMP-9 expression may be regulated in PSCs by Hic-5, a homologue of paxillin [134]. However, the direct mechanistic link between Hic-5 expression and MMP-9 is yet to be defined.

Undifferentiated monocytes in the PDAC TME can contribute to ECM degradation [135]. While in other tumor models, tumor-associated macrophages contribute to the breaching of the basement membrane by invasive tumor cells [136], in some contexts, monocytes can downregulate invadopodia formation in PDAC cells. For example, undifferentiated monocytes secrete the tissue inhibitor of metalloproteinase 2 (TIMP2, inhibitor of MT1-MMP), resulting in reduction of the PDAC cells invadopodial degradative capacity [137]. Beyond regulating the formation of invadopodia by tumor cells, macrophages can directly facilitate intravasation by tumor cells in other cancer types [136].

Altogether, these studies suggest that the PDAC TME has a dual effect by promoting and inhibiting invadopodia-mediated ECM degradation by cancer cells. Furthermore, these studies also demonstrate that CAFs can degrade the ECM by forming invadopodia and/or via novel degradative structures that are yet to be defined.

### 3.3. Invadopodia and Matrix Degradation: Final Thoughts

The studies discussed earlier in this section underscore the critical role that invadopodia and matrix degradation play in the early metastatic cascade. However, as noted previously, the study of invadopodia and matrix degradation each present major challenges. For instance, in addition to being highly dynamic, invadopodia are relatively small cellular structures that are difficult to capture in vivo. They would likely only be detected in actively invading cells. Furthermore, invadopodia are not ubiquitously found in all patient-derived PDAC cell lines in standard culture conditions, suggesting a similar heterogeneity among PDAC patients. Finally, tumor cells could accomplish ECM degradation through the non-invadopodial secretion of proteinases, including MMPs and even lysosomal proteases such as cathepsins. The relative contribution of invadopodial versus non-invadopodial ECM remodeling is not known.

From a therapeutic standpoint, the findings discussed herein raise multiple questions: (1) How can dynamic invadopodia be identified in vivo, particularly in patient tumor samples? (2) If invadopodia are not ubiquitous, on what basis would patients be stratified as invadopodia-positive versus invadopodia-negative for potential invadopodia-targeted therapies? (3) Since structures other than invadopodia can facilitate protease-mediated matrix degradation, how critical are invadopodia to metastasis and to what extent will patients benefit from invadopodia-targeted therapy? (4) Would it be more effective to target invadopodia found on cancer cells, or other degradative structures found on other cell types in the TME, such as CAFs? These are all critical questions that must be addressed if invadopodia are to be considered as a potential therapeutic target in the future. To this end, sufficient improvements will need to be made in imaging, as well as in identifying more specific markers of functional invadopodia and in our understanding of the relative contribution of invadopodia to cancer cell invasion, migration, and metastasis.

## 4. Tumor Cell Migration via Lamellipodia and Focal Adhesions

While invadopodia extend into the tumor stroma and degrade the ECM, tumor cells must also become motile in order to migrate towards the basement membrane and intravasate into the bloodstream. Cell motility is a complex phenomenon facilitated by multiple structures that engage with surrounding ECM proteins to accomplish cell movement, such as lamellipodia, focal complexes, and focal adhesions. Lamellipodia and filopodia are actin-based protrusions that couple membrane deformation with the cytoskeletal remodeling machinery to facilitate locomotion in cells with a mesenchymal migratory phenotype. These leading-edge protrusions form dynamic adhesions with the ECM called focal adhesions (FAs), which provide the mechanical force for migration and anchor the ECM to the cytoskeleton, while simultaneously responding to changes in the extracellular environment [138]. Focal adhesions rely on bi-directional integrin signaling to sense forces and transmit signals between the cell and ECM that can trigger a wide range of downstream signaling pathways with diverse functions, including cell proliferation, survival, and motility [139].

### 4.1. Lamellipodia and Filopodia in PDAC Cells

Lamellipodia and filopodia are driven by activation of the Rho family GTPases Rac and Cdc42, respectively, and require a host of actin regulatory proteins, including actin-binding, -bundling, and -capping proteins. In PDAC, multiple actin regulatory proteins are aberrantly expressed or activated to support the formation and action of lamellipodia and filopodia to promote migration.

Migratory actin dynamics are initiated by signals downstream of Rac and Cdc42, which are active in their GTP-bound state, activated by GEFs, and inhibited by GAPs. This dynamic cycle of activity is often amplified in PDAC to promote migration. For example, the inflammatory cytokine IL-6, known to be present and potent in PDAC, can promote Cdc42-mediated filopodia formation through the scaffolding protein IQGAP1 [140]. The GTPase Rab5A, which regulates vesicular trafficking, supports filopodia formation and migration in PDAC cell lines by promoting the activity of Cdc42 and the activation of β1-integrin [141]. As RhoGTPases, including Rac and Cdc42, are activated by GEFs, overexpression or hyperactivation of GEFs in PDAC can activate migration via promoting lamellipodial and filopodial-based migration. For example, the ectopic expression of the GEF Vav1 in PDAC promotes Rac-mediated lamellipodia formation and migration [142]. The Rac GEF Eps8, which also binds to actin directly, was found to be overexpressed in PDAC, to associate with actin in membrane ruffles and the tips of filopodia, and to promote PDAC cell migration [143]. Conversely, inhibition of Rac1 by GAPs can restrain actin remodeling and cell migration. The protein BART was found to bind and inhibit Rac1 in cell protrusions, potentially acting as a GAP, to restrict actin dynamics and PDAC cell migration [144].

Downstream of RhoGTPases, actin dynamics that drive lamellipodia and filopodia are similar to those in invadopodia formation, and include actin nucleation via Arp2/3, polymerization, capping to prevent new growth, and severing to initiate new sites for branching and polymerization. Multiple actin regulatory proteins are amplified or aberrantly turned on in PDAC. The large GTPase Dynamin 2, which links membrane deformation and the actin cytoskeleton, also regulates lamellipodial protrusion and dynamics to promote PDAC cell migration [102]. The actin-binding proteins TRIOBP4-5 were found to be upregulated in PDAC and to promote filopodia formation and cell motility [145]. The tankyrase-binding protein TNKS1BP1 had an unexpected role in regulating lamellipodial actin ruffling and dynamics by regulating the actin association of the capping protein CapZA2 [146]. Neuroligin 1 is a synaptic protein found to be expressed in PDAC, and it regulates filopodia formation and migration along nerves via regulating the activity of the actin-severing protein cofilin [147]. Thus, dysregulation of the expression or function of actin regulatory proteins in PDAC can promote the formation of migratory protrusions.

### 4.2. Focal Adhesion Dynamics in PDAC

**Figure 3:**
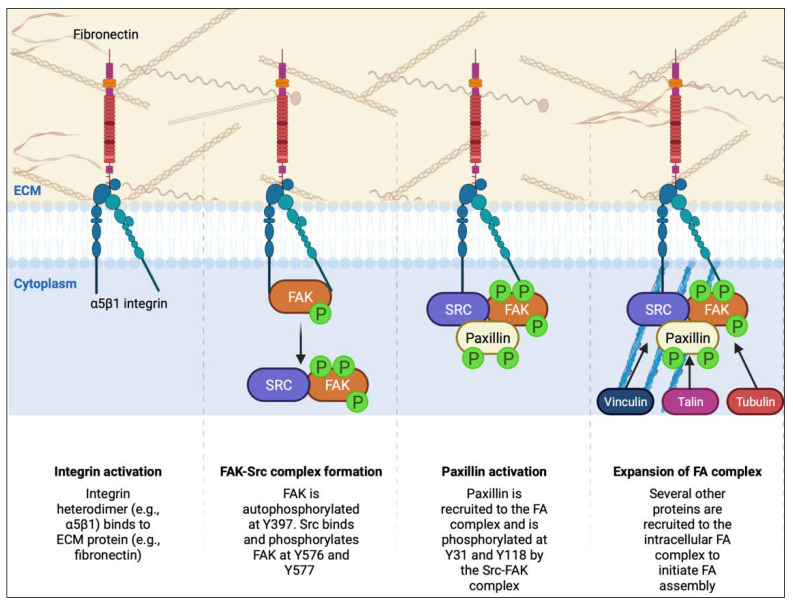
Focal adhesion initiation signaling. (ECM—extracellular matrix; FA—focal adhesion; FAK—focal adhesion kinase; Y—tyrosine). Integrins form dimers and interact with extracellular matrix proteins. Inside the cell, the action of focal adhesion kinase and Src facilitates the assembly of a nascent focal contact, which recruits and assembles a multi-protein complex including the focal adhesion proteins paxillin, talin, and vinculin. These factors bridge the connection to the cytoskeleton and facilitate mechanotransduction. Figure created on Biorender.com.

Migratory structures, including lamellipodia, are stabilized through adhesive interactions with the ECM called focal adhesions. Focal adhesion assembly and function require a complex machinery of proteins that are tightly regulated to modulate adhesion dynamics and drive cell motility (Figure 3). Conversely, focal adhesions must be disassembled to allow a cell to detach from the ECM and continue to translate through the ECM. The delicate balance between focal adhesion assembly and disassembly—focal adhesion dynamics—plays a key role in determining the migratory capacity of cells [148]. Highly stable focal adhesions facilitate strong linkages between the cell and ECM; however, such linkages can be a hindrance to rapid changes in cell morphology and cell motility.

Several key structural and regulatory proteins involved in focal adhesion dynamics are dysregulated in cancer to enhance cell invasion and migration, including integrins, FAK, Src kinase, paxillin, and the actin and microtubule cytoskeletons [149,150,151,152]. Focal adhesions facilitate connections to ECM proteins, which, in PDAC, include a rich desmoplastic ECM composed of type I collagen, type III collagen, type IV collagen, and fibronectin [153,154]. Given the complexity and density of the ECM and the role that PDAC cells play in promoting the deposition of ECM components, focal adhesions are of significant relevance in this disease context. In this section, we will examine recent advances in our understanding regarding focal adhesion dynamics and the dysregulation of key drivers of focal adhesion formation.

#### 4.2.1. Integrin Trafficking

Activation of integrin receptors is the first step in focal adhesion assembly. Integrins are transmembrane proteins that assemble in heterodimers to bridge the extracellular matrix and the actin cytoskeleton [155]. Integrins binding to their extracellular matrix ligands leads to their activation, which stimulates a conformational change leading to recruitment and clustering of focal adhesion proteins. Intracellular trafficking and recycling of integrins via the endocytic machinery has emerged in recent decades as a major mechanism of integrin regulation and expression at the cell surface. The existence of 24 integrin heterodimers and their unique involvement in a wide range of cell types, cellular contexts, and biological processes results in a broad diversity of trafficking and recycling mechanisms [155].

Integrins at the plasma membrane are trafficked to the cell surface via classical secretory trafficking and are then cleared from the cell surface by clathrin-mediated endocytosis. The integrins can subsequently be recycled to the plasma membrane or trafficked to the lysosome for degradation [156]. Recent studies have investigated mechanisms regulating integrin trafficking in PDAC and the consequences for focal adhesion dynamics and cell migration. For example, Li et al. reported that the GTPase RhoC binds to integrin α5β1 and significantly increases its internalization and recycling back to the plasma membrane [157]. Ectopic expression of RhoC in PDAC cells reduced paxillin staining and focal adhesion size in vitro, indicating increased focal adhesion turnover, and increased migration and invasion. In addition, RhoC-α5β1 co-localization was increased in primary PDAC tumors from mice and patients with distant metastases, suggesting that upregulation of RhoC-mediated α5β1 integrin trafficking and recycling increase PDAC metastasis in vivo [157].

α5β1 integrin trafficking and recycling has also been shown to be regulated by TGF-β signaling and autophagy. TGF-β treatment in vitro led to a significant increase in α5β1 integrin internalization and recycling, with reductions in activated FAK and paxillin. Mechanistically, TFEB promotes enhanced α5β1 turnover by transcriptionally upregulating the early endosomal RAB GTPase RAB5A, which then co-localizes with α5β1 in PDAC cells in vitro upon TGF-β treatment [158]. Autophagy may also enhance cancer cell migration by increasing α5β1 integrin recycling and focal adhesion turnover [158]. TGF-β treatment in PDAC cell lines induced autophagy via upregulation and enhanced nuclear translocation of the transcription factor EB (TFEB) [158].

#### 4.2.2. FAK and Paxillin Regulation

FAK and paxillin are critical promoters of focal adhesion assembly through their direct interaction, as well as their downstream signaling activity following activation. FAK is a tyrosine kinase with substrates involved in focal adhesions, including paxillin and p130Cas, and, most notably, FAK phosphorylates itself [159]. This recruits the oncogene SRC, which also phosphorylates FAK, and leads to focal adhesion turnover [160]. Paxillin is a multi-domain scaffold protein that is phosphorylated, and that recruits multiple focal adhesion proteins, including regulators of Rac and Cdc42 GTPases.

FAK and paxillin activity are highly regulated in PDAC cancer cell migration and invasion. Active FAK has been reported to be stabilized or upregulated in vitro by the receptor tyrosine kinase (RTK) EphA2, tissue transglutaminase (TG2), pancreatic adenocarcinoma upregulated factor (PAUF), and tetraspanin 8 (Tspan8), while being downregulated in vitro by PTEN and neutrophil gelatinase-associated lipocalin (NGAL) [161,162,163,164,165,166]. Similarly, paxillin can be upregulated by PAUF, Tspan8, and WNT5A [164,165,167]. However, technical limitations in some of these studies limit the ability to draw strong conclusions about the interactions of these proteins with FAK/paxillin, the functional impact of these potential regulators on focal adhesion dynamics, and where these potential regulators fit within the larger signaling network that regulates focal adhesions and cell migration in PDAC.

Liu et al. uncovered a novel pathway in PDAC involving ZIP4, ZEB1, ZO-1, and claudin-1 that upregulates the activation of both FAK and paxillin [168]. Overexpression of the transmembrane protein ZIP4 increased levels of the transcription factor ZEB1, which then transcriptionally repressed claudin-1 and ZO-1. As a result, phosphorylation of FAK and paxillin increase, leading to enhanced PDAC cell migration and invasion in vitro, as well as increased metastases in vivo [168]. It is unclear how ZO-1 and claudin-1 downregulate the activation of FAK and paxillin. Studies have shown that depletion of ZO-1 leads to enhanced activated FAK and activated paxillin in endothelial cells [169]. Loss of claudin-1 has been shown to have a dual effect in promoting and suppressing tumor progression across cancers [170], and depletion of claudin-1 increases PDAC tumor growth and metastasis [168]. Interestingly, these findings suggest a degree of overlap between regulators of EMT and focal adhesions; specifically, ZIP4 and ZEB1 play important roles in promoting both EMT and cancer cell migration and invasion [168,171]. Whether these signaling cascades are activated in concert, in a stepwise fashion, or independently of each other remains to be elucidated.

The small GTPase RhoA and its effector ROCK are key players in focal adhesion maturation. Following activation of the cholecystokinin B receptor (CCKBR) by its ligand gastrin, both of which are upregulated in PDAC [172,173,174], the Rho GTPase activators, Gα12 or Gα13 proteins, activate RhoA, which then activates its downstream effector ROCK, leading to the activation of FAK and paxillin. This results in gastrin-mediated enhancement of focal adhesion formation and F-actin assembly, as well as enhanced PDAC cell motility and invasion in vitro [172]. These findings were validated in vivo in a mouse model, wherein treatment with gastrin increased the number of liver metastases [172]. These results revealed a novel Gastrin-CCKBR-Gα12/13-RhoA-ROCK regulatory mechanism, which enhances cancer cell migration, invasion, and metastasis by promoting focal adhesion maturation [175].

Importantly, FAK inhibitors are proposed as promising therapeutic approaches for pancreatic cancers, showing inhibition not only of metastasis, but also tumor growth, stemness, therapy resistance, and interactions with the tumor microenvironment. Thus, FAK and other components of focal adhesions may have broad effects that contribute to their value as therapeutic targets.

#### 4.2.3. Microtubule Dynamics in Focal Adhesion Turnover

Microtubules (MTs) are dynamic cytoskeletal structures composed of α-tubulin and β-tubulin heterodimers that play a critical role in cellular functions, including intracellular trafficking of cargo, maintenance of cell shape, cell motility, and cell division [176]. In the last few decades, a clear role for MTs in focal adhesion dynamics has also emerged. MTs are directed towards mature focal adhesions to promote their disassembly, typically at the rear of moving cells, in order to promote continued cell migration [177]. Key proteins involved in focal adhesion dynamics are trafficked along microtubules to and from the membrane, including GTPases, proteins involved in actin polymerization, and vesicles responsible for integrin trafficking and recycling [178]. In addition, microtubules can both directly and indirectly promote endocytosis of integrins and affect acto-myosin contractility [177]. This may contribute to changes in focal adhesion turnover and facilitate cancer cell migration and invasion.

Recent studies have sought to determine whether microtubule dynamics are associated with focal adhesions and cancer cell motility. Dyn2 is a major regulator of microtubule dynamics, contributes to PDAC cell invasion, and has previously been shown to interact with FAK to promote focal adhesion disassembly via microtubules [179,180,181]. PODXL is a type 1 transmembrane protein that is overexpressed in PDAC and has been linked to a cell invasion [182,183,184]. Wong et al. demonstrated that PODXL binds and co-localizes with Dyn2 in vitro at focal adhesions, and that this interaction conferred a pro-migratory phenotype by enhancing the microtubule growth rate and downstream activation of Src kinase [185]. Thus, these findings uncovered a novel regulatory mechanism of focal adhesion dynamics in PDAC cells that promotes cancer cell migration independent of RhoA/Rac1 signaling and actin dynamics [185].

Kinesins are motor proteins that transport cargo along microtubules. In a screen of kinesins involved in integrin trafficking in PDAC cell lines, He et. al. identified KIF15 as a key regulator of integrin β1 recycling, FAK phosphorylation, and focal adhesion disassembly [186]. KIF15 interacts with integrin β1, PI3KC2, and Rab11, which marks recycling endosomes, and is regulated by its phosphorylation and acetylation to support tumor cell invasion [186].

Recent evidence also points to changes in cancer cell migration that are mediated through concomitant alterations in actin and microtubule dynamics through a common upstream regulator. Using human pancreatic duct epithelial (HDPE) cells, Tuntithavornwat et al. demonstrated that giant obscurin, a cytoskeletal protein mutated in 5–8% of PDAC tumors, regulates RhoA-mediated cytoskeletal remodeling in a tumor-suppressive capacity by downregulating cell motility, microtubule growth rate, and actin dynamics. Thus, depletion of giant obscurin in vivo promotes PDAC tumor growth and metastasis [187].

### 4.3. Role of the TME in PDAC Focal Adhesions

Focal adhesion dynamics depend on the bi-directional signaling of integrins and their engagement with the ECM, including collagens, fibronectin, and laminin [153,154]. Each of these ECM components binds to specific integrins on the cell surface and can influence the activation of specific signaling pathways, the pattern of integrin activity in PDAC cells, cell survival, motility, and proliferation. To create a pro-tumor microenvironment, PDAC cells actively contribute to ECM remodeling through crosstalk with other cell types in the TME. PSCs and CAFs deposit ECM proteins, such as collagen and fibronectin [154], which can activate integrin receptors and promote cell migration. While there are limited studies that have examined how CAFs and other cell types in the PDAC TME influence focal adhesion dynamics, there is evidence that PSCs/CAFs and PDAC cells do engage in crosstalk that affects key regulators of focal adhesions, such as FAK and paxillin, as well as the adhesive and migratory capacity of PDAC cells.

Lu et al. investigated how PSCs influence different types of cancer cell migration in vitro, including chemotaxis, chemokinesis, haptotaxis, and haptokinesis [188]. Using a co-culture model, the authors found that PSC supernatant (PSC-SN) induced haptotaxis or haptokinesis in PDAC cells and significantly enhanced adhesion and migration velocity. They also demonstrated that collagen I, secreted by PSCs, is the primary driver of the enhanced cell adhesion and motility. Furthermore, co-culture of PDAC cells with PSC-SN or on a collagen I matrix led to an increase in the phosphorylation of FAK and paxillin at the cell periphery, while FAK inhibition significantly abrogated haptokinesis and haptotaxis induced by PSC-SN or collagen I [188]. Although the authors did not directly examine focal adhesion dynamics, the changes in FAK and paxillin activation and the effect of FAK inhibition on the motility of PDAC cells suggest that adhesive molecules secreted by PSCs—primary collagen I—activate focal adhesion signaling molecules and enhance cell adhesion and motility [188]. There is also likely crosstalk between PDAC cells and other molecules from the PSC secretome that lead to enhanced focal adhesion turnover and facilitate cell motility.

In addition to ECM composition, differences in the organization pattern of adhesion molecules can influence the migratory and invasive abilities of PDAC cells. Both mouse and human PDAC tumors exhibit distinct tumor-associated collagen signatures (TACSs) that are characterized by unique densities, matrix stiffness, and spatial arrangements in relation to the tumor cells [189]. Using a 3D organoid model, Ray et al. demonstrated that specific TACSs facilitate enhanced single-cell invasion of PDAC cells into the surrounding stroma. Inhibition of FAK was associated with significantly fewer single-cell extrusions and lung metastases in an in vivo mouse model of the TACS-3 phenotype only. Due to the unique physical properties of each TACS phenotype, these findings underscore a role for FAK as a mechanotransducer [189].

While a pivotal role for FAK expression in cancer cell motility has long been established, recent evidence suggests that FAK expression in CAFs can also influence cancer cell migration. Mice orthotopically injected with PDAC cells and fibroblasts expressing kinase-inactive FAK (FAK-KD) had fewer metastases than fibroblasts with WT FAK [190]. In addition, FAK-KD mouse CAFs are oriented differently compared to FAK-WT mouse CAFs in relation to the tumor in vivo, are unable to deposit ‘tracks’ that tumor cells can follow during migration, are significantly less contractile in vitro, and, in the case of human FAK-KD CAFs, deposit fewer core ECM proteins, including collagens, glycoproteins, and proteoglycans. In addition, β1 integrin activation in PDAC cells was significantly reduced upon co-culture with human CAFs treated with a FAK inhibitor, suggesting decreased focal adhesion dynamics due to deficient ECM remodeling by CAFs lacking FAK activity. Taken together, these data implicate CAF FAK activity as a modulator of multiple processes that promote PDAC cell migration, invasion, and metastasis [190].

### 4.4. Focal Adhesions: Final Thoughts

Focal adhesions play a critical role in the early metastatic cascade by enabling cells to interact with and migrate through the ECM. As discussed, the heterogeneity found among integrins, as well as the various adhesion molecules that comprise the ECM, are consistent with the concept that focal adhesion dynamics can be regulated both intracellularly and extracellularly. In addition to the wide range of regulatory mechanisms modulating focal adhesion behavior, the dynamic nature of these structures also makes the study of focal adhesions challenging. Furthermore, it is evident that many upstream regulators of focal adhesion dynamics—such as FAK, Src kinase, and integrins—play multiple roles and, thus, are not highly specific to focal adhesions. From a clinical standpoint, this presents a challenge, as therapies targeted towards focal adhesions would require a high degree of specificity. Focal adhesions that enhance cancer cell migration are neither too stable nor too unstable—stable enough to adhere to the ECM, but also turned over quickly to enable the formation of new focal adhesions as the cell migrates. Thus, there is an unmet need to develop more sensitive and specific tools with which to study these dynamic structures, particularly in vivo, in order to better understand their contribution and regulation during cancer cell migration and metastasis.

## 5. Conclusions

Improving patient outcomes for PDAC will require understanding and halting metastatic dissemination. While metastasis has often been correlated with EMT, recent evidence demonstrated that EMT in PDAC exists on a spectrum of states that are not yet fully elucidated. Furthermore, data suggest that classical transcriptional EMT may not be necessary for metastatic dissemination in PDAC, and its role in metastasis largely depends on the cellular context, such as the mode of migration. Thus, while crucial, EMT is only one aspect of metastatic cell biology.

The formations of functional invadopodia, lamellipodia, and focal adhesions are important mechanisms by which PDAC cells invade the surrounding matrix, cross the basement membrane, and navigate the dense stroma. Invadopodia and focal adhesions are both highly dynamic structures that are difficult to discern in vivo; furthermore, other processes and cell types in the TME also contribute to matrix degradation. The contributions of invadopodial versus non-invadopodial ECM degradation in PDAC metastasis, as well as of ECM degradation mediated by other cell types in the TME, remain to be fully elucidated. An incomplete understanding of the regulation of EMT, invadopodia, and focal adhesions, combined with technological limitations to study these processes in vivo, prevents a comprehensive understanding of early metastatic dissemination. Therefore, future studies should focus on further elucidating the regulatory mechanisms underlying cancer cell migration and invasion, understanding how these mechanisms overlap, finding unique markers that can enable high-resolution identification of structures such as invadopodia and focal adhesions in vivo, and, importantly, contextualizing these findings in the broader TME by considering the impact of other cell types and structures on the tumor’s ability to metastasize.
